# Interaction Between Smoking and Olfactory Function on Frailty: A Population‐Based Cross‐Sectional Study

**DOI:** 10.1002/hsr2.71266

**Published:** 2025-10-28

**Authors:** Guangyao Li, Fangzhou Ye, Keguang Chen

**Affiliations:** ^1^ Department of Otorhinolaryngology – Head and Neck Surgery Shanghai Zhongshan Hospital Affiliated to Fudan University Shanghai PR China; ^2^ Department of Otolaryngology Eye & ENT Hospital Fudan University Shanghai PR China

**Keywords:** frailty, NHANES, olfactory impairment, smoking

## Abstract

**Background and Aims:**

Frailty is a multidimensional syndrome characterized by decreased physiological reserves and is closely associated with aging‐related adverse outcomes. Although smoking and olfactory dysfunction are individually associated with frailty, their potential interactions have not been thoroughly investigated.

**Methods:**

We used data from the National Health and Nutrition Examination Survey (NHANES) 2011–2014, which included 5192 participants. Smoking status and self‐reported olfactory function were assessed using standardized questionnaires. Frailty was defined using a 36‐item Frailty Index (FI), with FI > 0.2 indicating frailty. Multivariate logistic regression models were used to examine the independent and joint associations of smoking and olfactory function with frailty, after adjusting for demographic and lifestyle covariates. Interaction and stratified analyses were also performed.

**Results:**

Current smoking status and altered olfactory function were independently associated with an increased risk of frailty. In fully adjusted models, nonsmokers had a lower odds of frailty compared with smokers (odds ratio [OR] = 0.41, 95% confidence interval [CI]: 0.29–0.58), and participants with normal olfactory function had a lower odds of frailty than those with altered olfactory function (OR = 0.57, 95% CI: 0.48–0.69). A significant interaction was observed between smoking and olfactory function (*P* interaction = 0.001). The highest odds of frailty were found in participants who smoked and had altered olfactory function (OR = 2.49, 95% CI: 1.88–3.31).

**Conclusion:**

Our findings revealed a synergistic association between smoking, altered olfactory function, and frailty risk. This interaction in relation to the healthcare of older people highlights the importance of sensory screening and behavioral risk assessment in the early identification of frailty, especially in smokers.

## Abstract (highlights)

**Summary:**

- Smoking and altered olfactory function were independently associated with an increased risk of frailty.
- A significant interaction was observed between smoking and olfactory function, suggesting a stronger effect of olfactory alterations on the frailty risk among smokers than among nonsmokers.
- Participants with both risk factors—altered olfactory function and current smoking—had the highest likelihood of frailty.

**Why does this paper matter?:**

With regards to the healthcare of older people, the interaction between smoking and altered olfactory function highlights the importance of sensory screening and behavioral risk assessment in the early identification of frailty, especially in smokers.

## 1 Introduction

Frailty is a syndrome characterized by progressive decline in multiple physiological systems, resulting in increased vulnerability to external stressors and adverse health outcomes, including disability, hospitalization, and mortality [1, 2]. As the global population continues to age, frailty has emerged as one of the most pressing public health challenges of the 21st century [3]. Importantly, frailty is not an inevitable consequence of aging; it is often preventable, and in many cases, reversible during the early stages. Frailty is irreversible only during the terminal phase. Therefore, identifying modifiable risk factors and implementing strategies to delay or prevent the onset of frailty are critical for improving health outcomes in older adults [4].

Cigarette smoking is a well‐established modifiable risk factor that contributes to a host of chronic diseases and conditions, including frailty, in older adults. Several mechanisms by which smoking exacerbates frailty have been proposed. For instance, smoking is implicated in the generation of oxidative stress and systemic inflammation, both of which can cause deterioration of physiological function and nutritional status [5, 6]. A systematic review and meta‐analysis indicated that current smokers were significantly more likely to exhibit frailty than nonsmokers, particularly in older populations [7]. Additionally, a large‐scale cohort study demonstrated that individuals who reported past smoking did not exhibit a greater risk of incident frailty than never smokers, implying the potential benefits of smoking cessation [8].

Evidence suggests that sensory impairments, particularly olfactory dysfunction, may be early indicators of physiological decline and may increase the risk of frailty [9, 10, 11, 12, 13]. While several studies have independently linked smoking and olfactory dysfunction to frailty, few have examined their combined effect. One large population‐based analysis found that chronic smoking was associated with a higher likelihood of self‐reported olfactory decline, suggesting a biological interaction that may further contribute to frailty risk [14]. However, direct investigations into how smoking may exacerbate the impact of olfactory dysfunction on frailty remain limited. By examining these relationships in a nationally representative sample and adjusting for relevant sociodemographic and health‐related factors, this study aimed to provide novel insights into the role of sensory and behavioral risk factors in the development of frailty and to inform early identification strategies for vulnerable populations.

Given that smoking is modifiable and olfactory impairment is detectable at an early stage, exploring their interaction may offer critical perspectives for frailty prevention and risk stratification. Therefore, in this study, we aimed to investigate the independent and joint associations between smoking status, olfactory function, and frailty among adults in the United States using data from the National Health and Nutrition Examination Survey (NHANES) 2011–2014. We hypothesized that both smoking and impaired smell would be independently associated with a higher frailty risk and that their combination would exhibit an interaction effect, further amplifying this risk.

## 2 Methods

### 2.1 Study Population

The NHANES is a continuous cross‐sectional survey designed to assess the health and nutritional status of the noninstitutionalized civilian population in the United States. It uses a complex multistage probability sampling design that incorporates interviews, physical examinations, and laboratory assessments.

**Figure 1:**
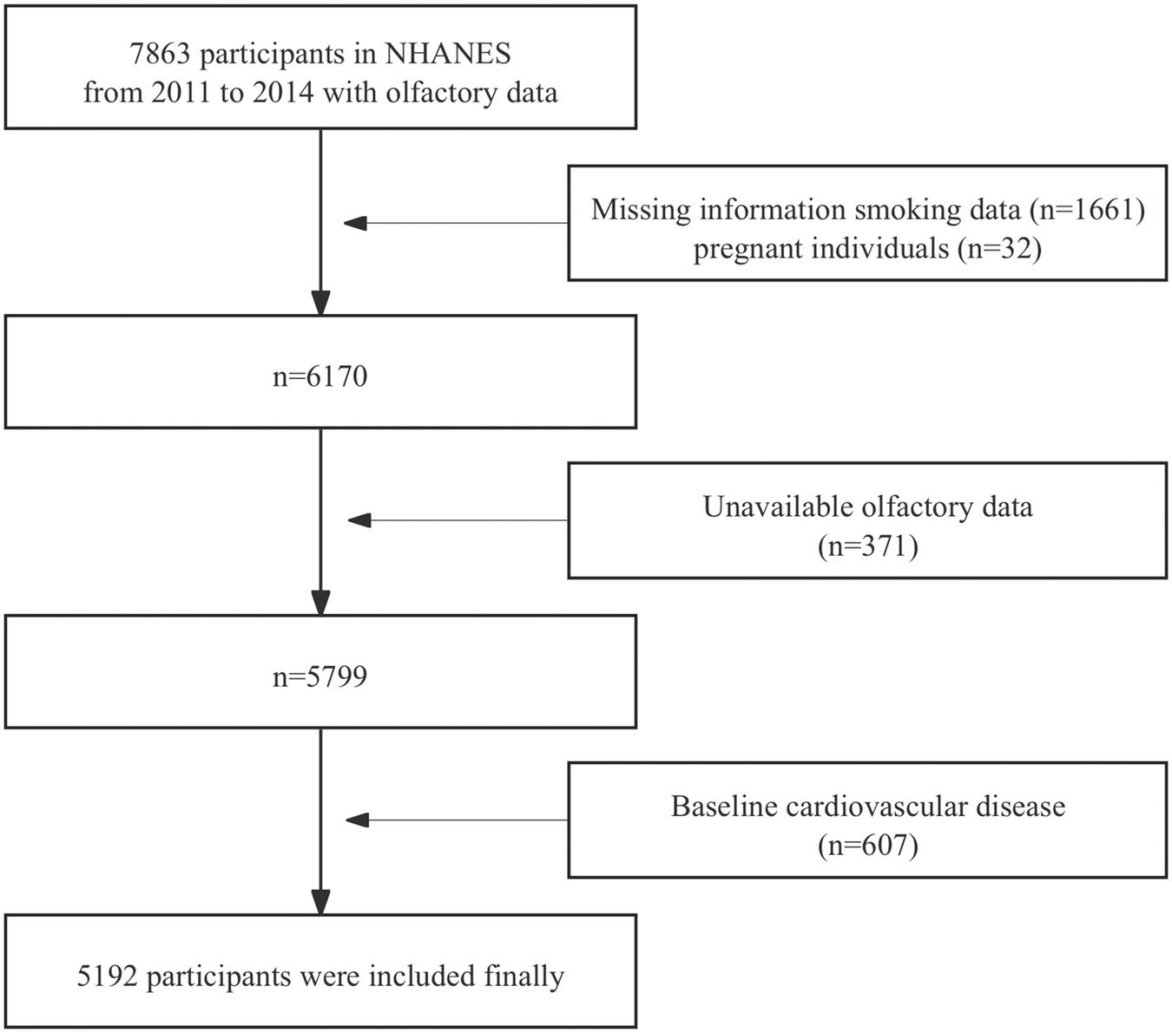
Flowchart of the study design and participants.

Data were extracted from the NHANES cycles conducted between 2011 and 2014 (two cycles), during which self‐reported olfactory function was collected through a questionnaire. A total of 7863 participants provided responses regarding olfactory function. As illustrated in Figure 1, the following exclusion criteria were applied: (1) individuals with missing information on smoking status (*n* = 1661) or those who were pregnant (*n* = 32); (2) participants with unavailable self‐reported olfactory data (*n* = 371); and (3) individuals with a self‐reported history of baseline cardiovascular disease (*n* = 607). A total of 5192 participants aged 40–80 years met the inclusion criteria and were retained for the final analysis. After applying NHANES sampling weights, the weighted mean age was 56.9 ± 11.1 years; the corresponding unweighted mean age was 58.2 ± 11.7 years.

### 2.2 Assessment of Frailty

In this study, frailty was evaluated using a 36‐item Frailty Index (FI) [15], adapted from a previously validated FI developed for use in NHANES populations (Table S1). The FI is calculated as the ratio of the number of health deficits present to the total number of deficits considered, yielding a continuous score ranging from 0 to 1. A higher FI indicates a greater degree of frailty.

Based on the established cutoff points, participants were initially categorized into four groups: fit (FI ≤ 0.1), vulnerable (0.1 < FI ≤ 0.2), mildly frail (0.2 < FI ≤ 0.3), and moderately/severely frail (FI > 0.3). For the purposes of statistical analysis, these categories were dichotomized: individuals with FI ≤ 0.2 were defined as nonfrail, and those with FI > 0.2 were considered frail, consistent with previous studies.

### 2.3 Assessment of Olfactory Function and Smoking Status

The NHANES olfactory questionnaire includes items on self‐reported olfactory ability, symptoms, medical treatment, and the presence of related risk factors for olfactory dysfunction. These questions were content‐validated by experts in olfaction and tested to ensure consistency in participants' understanding, processing, and interpretation. The questionnaire items were used to classify olfactory alterations as perceived changes in function since the age of 25 years [altered now] [16]. Positive responses to this question resulted in positive olfactory alteration scores. A dichotomous measure [“yes” or “no”] for olfactory alterations was the outcome variable in the data analyses. This classification has shown excellent test–retest reliability over 6 months and good correspondence with clinically supported risk factors [16, 17].

The NHANES home interview included questions about daily cigarette use, history of use, duration of smoking, and the time to first cigarette consumption upon waking in the morning. Smokers were classified based on an affirmative response to having ever smoked 100 cigarettes in their lifetime; never smokers answered “no.”

### 2.4 Covariates

Body mass index (BMI) was categorized into four groups based on standard clinical cutoffs: (1) underweight/healthy weight (BMI < 25.0 kg/m^2^), (2) overweight (25.0 ≤ BMI < 30.0 kg/m^2^), (3) obese (30.0 ≤ BMI < 40.0 kg/m^2^), and (4) severely obese (BMI ≥ 40.0 kg/m^2^). Race/ethnicity was grouped into two categories: non‐Hispanic White and other (Mexican Americans, other Hispanic, non‐Hispanic Black, and other races).

Sleep health was assessed based on participants' self‐reported average sleep duration per night. Sleep duration was categorized into three groups: (1) short sleep (< 6 or ≥ 10 h), (2) suboptimal sleep (6 to < 7 h), and (3) normal sleep (7 to < 10 h), according to prior research and NHANES categorization standards [18].

Alcohol consumption was classified according to NHANES definitions, based on the number of days participants reported consuming alcohol over the past 12 months. The participants were categorized into four groups: (1) nondrinkers, < 50 drinking days/year; (2) low‐frequency drinkers, 51–100 days/year; (3) moderate‐frequency drinkers, 101–200 days/year; and (4) high‐frequency drinkers, > 200 days/year.

Physical activity (PA) was assessed based on self‐reported minutes of moderate or vigorous physical activity per week. Participants were categorized into two groups: (1) inactive, engaged in < 90 min of moderate or vigorous PA per week; and (2) active, engaged in 90 min or more of moderate or vigorous PA per week [19].

### 2.5 Ethics Approval and Consent to Participate

The NHANES protocol was approved by the Institutional Review Board of the National Center for Health Statistics. Written informed consent was obtained from each participant before participation in the study. ID: NCHS IRB/ERB Protocol #2011‐12.

### 2.6 Statistical Analysis

To account for the complex, multistage sampling design of the NHANES and ensure population‐level representativeness, sample weights were applied as recommended by the National Center for Health Statistics. Sample weight (FINALWEIGHT) was used to construct survey design variables. Descriptive statistics were weighted to reflect the United States population. Descriptive statistics for continuous variables were summarized using medians and interquartile ranges (IQRs) due to non‐normal distribution, and group comparisons were assessed using the Wilcoxon rank‐sum test. Categorical variables were presented as unweighted frequencies and weighted percentages, with between‐group comparisons performed using the *χ* ^2^ test. Binary logistic regression models were constructed to evaluate the independent and joint associations between smoking status, olfactory function, and frailty, adjusting for relevant sociodemographic and lifestyle covariates, because the outcome (frailty) was dichotomous. Missing data were handled using complete case analysis. Sensitivity analysis excluding variables with > 10% missing data showed consistent results. Model 1 was unadjusted; Model 2 adjusted for age, sex, race/ethnicity, and BMI; and Model 3 further adjusted for sleep duration, alcohol consumption, and physical activity. Odds ratios (ORs) and 95% confidence intervals (CIs) were reported to reflect effect sizes and the precision of estimates. Statistical significance was determined using two‐sided tests with an alpha threshold of 0.05. To assess potential effect modification, multiplicative interaction terms (e.g., smoking × olfactory function) were introduced into regression models, and stratified analyses were conducted. Model robustness was assessed via sensitivity analyses and stratified regression. However, external validation was not conducted due to data set constraints. No correction for multiple comparisons was applied, as the primary analyses were prespecified. Variance inflation factors were used to evaluate multicollinearity, and the Hosmer–Lemeshow test assessed model calibration.

All analyses were conducted using IBM SPSS Statistics (Version 26.0, IBM Corp., Armonk, NY) and R software (version 4.1.0). The reporting of statistical analyses follows the Statistical Analyses and Methods in the Published Literature (SAMPL) guidelines [20] and incorporates recommendations from Assel et al. [21] for statistical reporting in clinical research.

## 3 Results

A total of 5192 participants were included in the final analysis, comprising 75.1% (3897/5192) nonfrail individuals and 24.9% (1295/5192) classified as frail based on the FI. As shown in Table 1, the frail group was significantly older than the nonfrail group (mean age: 58 vs. 55 years; *p* < 0.001) and had a higher BMI (mean BMI: 31.72 vs. 28.68 kg/m^2^; *p* < 0.001). They were also more likely to be female (66.3% vs. 51.5%), physically inactive (62.2% vs. 37.1%), or current smokers (58.3% vs. 45.4%), with all differences statistically significant (*p* < 0.001).

**Table 1:** Baseline characteristics of participants.

Characteristic	Level	Frailty Index (FI)		*p* value
		≤ 0.2	> 0.2	
Weighted *N* (%)		86,711,116.0 (79.4)	22,436,037.0 (20.6)	
Unweighted *n* (%)		3897 (75.1)	1295 (24.9)	
Sex (%)	Male	42,088,563.0 (48.5)	7,560,010.0 (33.7)	< 0.001
	Female	44,622,553.0 (51.5)	14,876,027.0 (66.3)	
Age (years)		55 (47, 64)	58 (51, 68)	< 0.001
Age groups (y) (%)	< 65	67,113,652.0 (77.4)	15,373,559.0 (68.5)	< 0.001
	65–80	16,400,520.0 (18.9)	4,859,390.0 (21.7)	
	≥ 80	3,196,944.0 (3.7)	2,203,088.0 (9.8)	
BMI (kg/m^2^)		28.68 (6.08)	31.72 (7.89)	< 0.001
BMI groups (%)	Severely obese	10,977,017.0 (12.7)	6,143,354.0 (27.4)	< 0.001
	Obese	19,526,190.0 (22.5)	5,856,492.0 (26.1)	
	Overweight	32,234,474.0 (37.2)	6,516,412.0 (29.0)	
	Underweight/healthy weight	23,973,435.0 (27.6)	3,919,779.0 (17.5)	
Race and ethnicity (%)	Non‐Hispanic White	64,614,961.0 (74.5)	15,191,976.0 (67.7)	< 0.001
	Other race	22,096,155.0 (25.5)	7,244,061.0 (32.3)	
Drinking status (%)	Nondrinkers	12,207,405.0 (14.1)	5,443,370.0 (24.3)	< 0.001
	Low‐frequency drinkers	29,935,089.0 (34.5)	6,830,721.0 (30.4)	
	Moderate‐frequency drinkers	14,012,686.0 (16.2)	2,577,368.0 (11.5)	
	High‐frequency drinkers	30,555,936.0 (35.2)	7,584,578.0 (33.8)	
Smoking status (%)	Smoker	39,353,130.0 (45.4)	13,081,222.0 (58.3)	< 0.001
	Nonsmoker	47,357,986.0 (54.6)	9,354,815.0 (41.7)	
Sleep health (%)	Short sleep	9,939,518.0 (11.5)	6,007,441.0 (26.8)	< 0.001
	Suboptimal sleep	24,141,734.0 (27.8)	6,524,909.0 (29.1)	
	Normal sleep	52,629,864.0 (60.7)	9,903,687.0 (44.1)	
PA (%)	Inactive	32,210,967.0 (37.1)	13,954,864.0 (62.2)	< 0.001
	Active	54,500,149.0 (62.9)	8,481,173.0 (37.8)	
Olfactory function (%)	Altered	16,860,715.0 (19.4)	6,835,618.0 (30.5)	< 0.001
	Normal	69,838,340.0 (80.6)	15,600,419.0 (69.5)	

Abbreviation: PA, physical activity.

Additionally, the prevalence of altered olfactory function was markedly higher in the frail group than in the nonfrail group (30.5% vs. 19.4%; *p* < 0.001), as was the proportion of participants with short sleep duration (defined as < 6 h/night; 26.8% vs. 11.5%; *p* < 0.001).

As presented in Table S2, 20.9% of the participants (1084/5192) self‐reported altered olfactory function. Compared to individuals with normal olfactory perception, those with altered olfaction were older (age groups ≥ 65 (y): 73.2% vs. 68.6%; *p* = 0.034), had a higher BMI (30.03 vs. 29.10 kg/m^2^; *p* = 0.010), and were more likely to be current smokers (55.1% vs. 46.1%; *p* = 0.002). A significant difference in race/ethnicity distribution was also observed (*p* = 0.020), while no statistically significant differences were found in physical activity or sleep duration.

Table 2 presents the results of logistic regression analyses examining the associations between smoking status, olfactory function, and frailty across three models with progressive covariate adjustment. In the unadjusted model (Model 1), both nonsmoking and normal olfactory function were significantly associated with lower odds of frailty. Compared with smokers, nonsmokers had an OR of 0.57 (95% CI: 0.43–0.74; *p* < 0.001). Similarly, participants with normal olfactory function had significantly lower odds of frailty than those with altered olfaction (OR = 0.57; 95% CI: 0.47–0.68; *p* < 0.001). These associations remained robust after adjusting for demographic variables in Model 2 (age, sex, race, and BMI). In the fully adjusted model (Model 3), which additionally controlled for sleep duration, drinking status, and physical activity, nonsmokers had 59% lower odds of frailty (OR = 0.41; 95% CI: 0.29–0.58; *p* < 0.001) compared with smokers. Likewise, normal olfactory function was associated with a 43% lower odds of frailty (OR = 0.57; 95% CI: 0.48–0.69; *p* < 0.001).

**Table 2:** Logistic regression analysis of frailty by smoking status and olfactory function.

	Model 1^a^		Model 2^b^		Model 3^c^	
	OR (95% CI)	*p*	OR (95% CI)	*p*	OR (95% CI)	*p*
Smoking status [reference = smoker]	0.57 (0.43–0.74)	< 0.001	0.46 (0.34–0.61)	< 0.001	0.41 (0.29–0.58)	< 0.001
Olfactory function [reference = altered]	0.57 (0.47–0.68)	< 0.001	0.54 (0.45–0.66)	< 0.001	0.57 (0.48–0.69)	< 0.001

^a^ Model 1 without adjustments.

^b^ Model 2 additionally adjusted for sex (male, female), age (year), race (non‐Hispanic White, other), body mass index (underweight/healthy weight, overweight, obese, severely obese).

^c^ Model 3 additionally adjusted for sleep health (short sleep, suboptimal sleep, normal sleep), drinking status (nondrinker, low‐frequency drinker, moderate‐frequency drinker, high‐frequency drinker), and physical activity (inactive, active).

**Figure 2:**
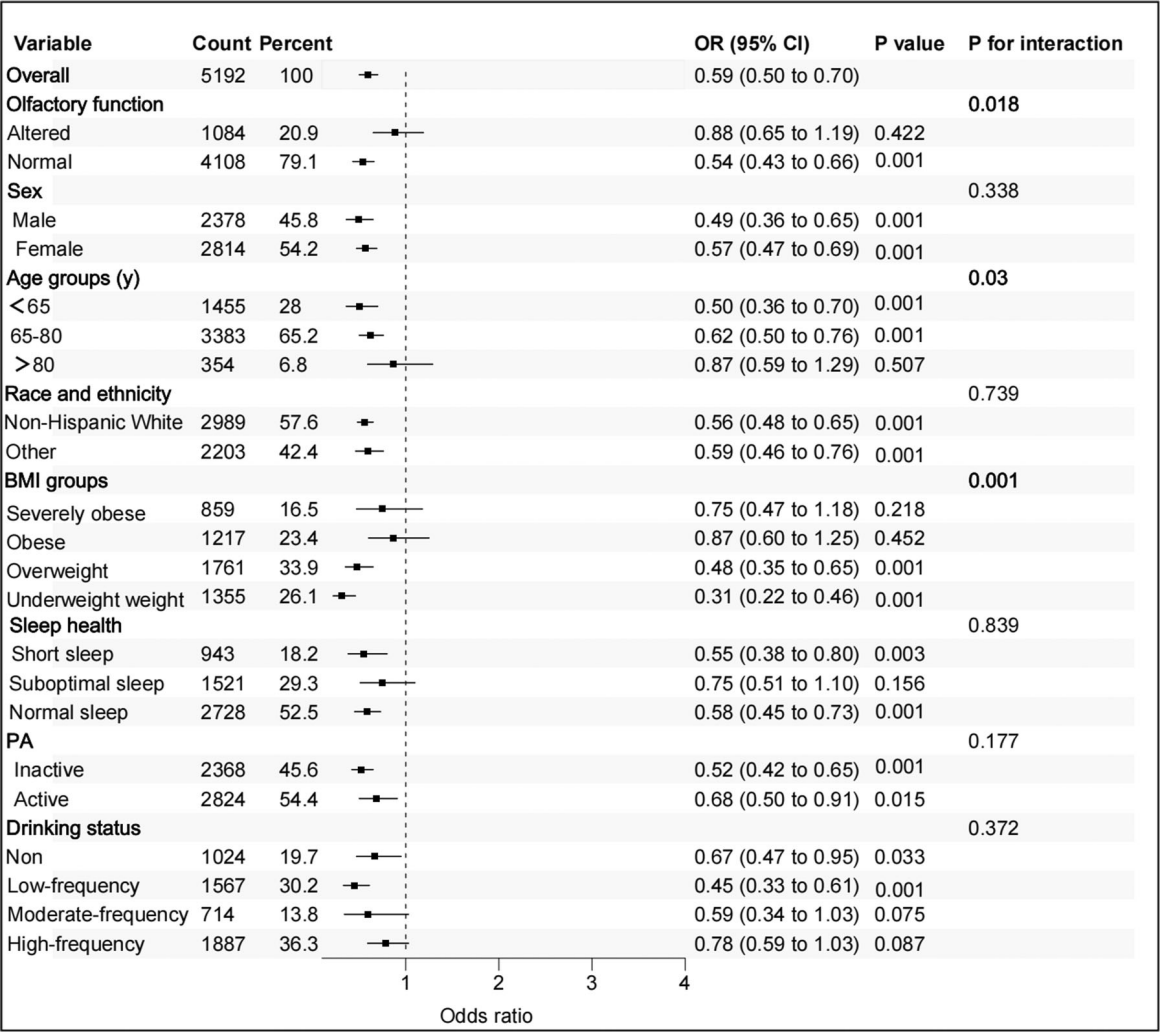
Forest plots of stratified analyses of the associations between smoking and frailty. Adjusted for sex (male, female), age (year), race (non‐Hispanic White, other), BMI (underweight/healthy weight, overweight, obese, severely obese), sleep health (short sleep, suboptimal sleep, normal sleep), drinking status (nondrinker, low‐frequency drinker, moderate‐frequency drinker, high‐frequency drinker) and physical activity (inactive, active). The variables examined in this table were not adjusted. BMI, body mass index; PA, physical activity.

We further explored the association between olfactory function and frailty through stratified analyses, and assessed the potential interaction effects with smoking status. As shown in Figure 2, a statistically significant interaction was observed between olfactory function and smoking status (*P* interaction = 0.018). In participants who smoked, normal olfactory function was significantly associated with a lower risk of frailty (OR = 0.54, 95% CI: 0.43–0.66, *p* < 0.001), whereas this association was not significant among nonsmokers. These findings suggest that the impact of olfactory dysfunction on frailty may be modified by smoking. To further explore this interaction, we categorized the participants into four subgroups based on their smoking and olfactory function status: (1) normal olfactory function and nonsmoker status, (2) normal olfactory function and smoking, (3) altered olfactory function and nonsmoking status, and (4) altered olfactory function and smoking. As presented in Table 3, and using Subgroup 1 as the reference, the adjusted ORs (Model 3) of frailty were: 1.78 (95% CI: 1.40–2.26) for Subgroup 2, 2.23 (1.62–3.06) for Subgroup 3, and 2.49 (1.88–3.31) for Subgroup 4 (*p* < 0.001 for all).

**Table 3:** Logistic regression analysis of frailty according to olfactory function and smoking status.

	Model 1^a^		Model 2^b^		Model 3^c^	
	OR (95% CI)	*p*	OR (95% CI)	*p*	OR (95% CI)	*p*
Subgroup 1	1.0 [reference]		1.0 [reference]		1.0 [reference]	
Subgroup 2	1.75 (1.51–2.03)	< 0.001	2.23 (1.75–2.80)	< 0.001	1.78 (1.40–2.26)	< 0.001
Subgroup 3	2.17 (1.75–2.71)	< 0.001	2.28 (1.71–3.05)	< 0.001	2.23 (1.62–3.06)	< 0.001
Subgroup 4	2.59 (2.12–3.16)	< 0.001	3.07 (2.36–3.99)	< 0.001	2.49 (1.88–3.31)	< 0.001

*Note:* Subgroup 1 is normal olfactory function and nonsmoker; Subgroup 2 is normal olfactory function and smoker; Subgroup 3 is altered olfactory function and nonsmoker; and Subgroup 4 is altered olfactory function and smoker.

^a^ Model 1 without adjustments.

^b^ Model 2 additionally adjusted for sex (male, female), age (year), race (non‐Hispanic White, other), and body mass index (underweight/healthy weight, overweight, obese, severely obese).

^c^ Model 3 additionally adjusted for sleep health (short sleep, suboptimal sleep, normal sleep), drinking status (nondrinker, low‐frequency drinker, moderate‐frequency drinker, high‐frequency drinker), and physical activity (inactive, active).

Stratified logistic regression analyses were performed to explore the robustness of the observed associations. As shown in Table S3, among participants who smoked, altered olfactory function was significantly associated with a higher risk of frailty. In the fully adjusted model (Model 3), the OR of frailty for altered versus normal olfactory function was 1.41 (95% CI: 1.09–1.81, *p* = 0.011). However, as shown in Table S4, smoking status was not significantly associated with frailty in the fully adjusted model among participants with altered olfactory function. The OR for smokers versus nonsmokers was 1.12 (95% CI: 0.80–1.57, *p* = 0.503).

## 4 Discussion

This study provides new evidence that both smoking and altered olfactory function are independently associated with frailty, and that their co‐occurrence confers a higher risk than either factor alone. These findings align with and extend previous research linking behavioral and sensory impairments to systemic vulnerability in aging populations.

Several biological and behavioral mechanisms may explain the observed associations between smoking, altered olfactory function, and increased risk of frailty [14, 22]. Previous studies have consistently demonstrated the role of smoking in promoting chronic inflammation, oxidative stress, and immune dysregulation, all of which contribute to physiological decline and frailty development [23, 24, 25, 26, 27, 28, 29]. Similarly, olfactory dysfunction has been identified as a predictor of cognitive impairment, depression, and malnutrition—key frailty‐related conditions [30, 31, 32, 33]. Analysis of olfactory impairment severity and health indicators revealed that both hyposmia and anosmia were significantly associated with increased odds of olfactory dysfunction. However, no significant associations were observed with other health indicators or economic outcomes [34]. Our findings suggest that these two risk factors may not only act independently but also interactively, amplifying their detrimental effects on functional health. This is consistent with the sensory‐accelerated aging model, in which environmental exposures such as tobacco use exacerbate sensory deterioration and its systemic consequences.

Our study's reliance on self‐reported olfactory changes, while practical for large‐scale surveillance, may have introduced misclassification. However, prior research indicates that self‐reported olfactory impairment is significantly associated with adverse clinical outcomes and may serve as a useful screening tool [35]. Identifying sensory decline in clinical or public health settings may offer valuable opportunities for early frailty screening and targeted interventions.

This study has some limitations. First, the cross‐sectional design prevented us from drawing causal conclusions. Second, olfactory function was assessed through self‐report rather than objective testing, which may have introduced misclassification. Nonetheless, self‐reported olfactory changes corresponded well with clinically relevant outcomes in previous studies. While we categorized smoking status based on the established NHANES definitions, we did not further stratify participants by dependence level, current smoking activity, or cumulative exposure indices (e.g., Brinkman Index). This decision was based on the primary aim of this study, which was to assess the synergistic effect of olfactory dysfunction and smoking status on frailty risk, rather than the isolated effect of smoking. Future studies focusing exclusively on tobacco‐related frailty risk may benefit from incorporating these detailed exposure parameters.

Importantly, our results underscore the potential clinical value of dual‐risk profiling. While smoking is a modifiable behavior, olfactory impairment may serve as an early, noninvasive biomarker of systemic decline. Integrating sensory screening into routine care—especially for smokers—may help identify individuals at elevated frailty risk and enable timely preventive interventions.

In conclusion, our findings highlight the synergistic impact of smoking and olfactory dysfunction on frailty. The interaction between these two factors suggests that olfactory dysfunction may serve as an early marker of vulnerability, particularly among smokers.

## Author Contributions

**Guangyao Li:** conceptualization, data curation, formal analysis, methodology, validation, visualization, writing – original draft. **Fangzhou Ye:** validation, writing – review and editing. **Keguang Chen:** project administration, resources, supervision, writing – review and editing. All authors have read and approved the final version of the manuscript.

## Ethics Statement

The NHANES protocol was approved by the Institutional Review Board of the National Center for Health Statistics, ID: NCHS IRB/ERB Protocol #2011‐12.

## Consent

Written informed consent was obtained from each participant before participation in the study.

## Conflicts of Interest

The authors declare no conflicts of interest.

## Transparency Statement

The lead author Keguang Chen affirms that this manuscript is an honest, accurate, and transparent account of the study being reported; that no important aspects of the study have been omitted; and that any discrepancies from the study as planned (and, if relevant, registered) have been explained.

## Supporting information


**Supplementary Table S1:** 36‐item Frailty Index.
**Supplementary Table S2:** Baseline characteristics of participants.
**Supplementary Table S3:** Logistic regression analysis of frailty by olfactory function among smokers.
**Supplementary Table S4:** Logistic regression analysis of frailty by smoking status among participants with altered olfactory function.

## Data Availability

The authors confirm that the data supporting the findings of this study are available within the article and its Supporting Information. The original NHANES data sets are publicly available at: !https://www.cdc.gov/nchs/nhanes/. Dr. Keguang Chen had full access to all of the data in this study and takes complete responsibility for the integrity of the data and the accuracy of the data analysis.
