# Supplementary material for: Interaction Between Smoking and Olfactory Function on Frailty: A Population‐Based Cross‐Sectional Study
Source: Health Sci Rep. 2025 Oct 28;8(10):e71266. doi: 10.1002/hsr2.71266 (PMC12559809; doi:10.1002/hsr2.71266)
Supplement: Supplementary file 1 — Supplementary Table S1: 36‐item Frailty Index. Supplementary Table S2: Baseline characteristics of participants. Supplementary Table S3: Logistic regression analysis of frailty by olfactory function among smokers. Supplementary Table S4: Logistic regression analysis of frailty by smoking status among participants with altered olfactory function. [file HSR2-8-e71266-s001.docx]

**Supplementary Material**

**Interaction between smoking and olfactory function on frailty: a population-based cross-sectional study**

Guangyao Li,^1^ Fangzhou Ye,^2^ Keguang Chen^1^*

^1^Department of Otorhinolaryngology-Head and Neck Surgery, Shanghai Zhongshan Hospital Affiliated to Fudan University, Shanghai 200032, PR China

^2^Department of Otolaryngology, Eye & ENT Hospital, Fudan University, Shanghai 200031, PR China

***Corresponding author**:

Keguang Chen

Department of Otorhinolaryngology-Head and Neck Surgery, Shanghai Zhongshan Hospital Affiliated to Fudan University

No.180 Fenglin Road, Xuhui District, Shanghai 200032, PR China

Phone:86+19370551995

Email: 22111260027@m.fudan.edu.cn

ORCID: [0009-0008-4694-4679]

**Supplementary Table S1.** 36-item Frailty Index

| Self-reported Frailty Index items | Laboratory Frailty Index items |
| --- | --- |
| 1. Angina/angina pectoris | 26. Pulse rate (60-99 bpm) |
| 2. Heart attack | 27.Systolic blood pressure (90-140mmHg) |
| 3. Coronary heart disease | 28. Pulse pressure (30-60 mmHg) |
| 4. Stroke | 29. Platelet count SI (150-450 unit 1000  cells/mL) |
| 5. Thyroid condition | 30. Blood urea nitrogen (3-20 mg/dL) |
| 6. Cancer | 31. Bicarbonate (≤28 mmol/L) |
| 7. Arthritis | 32. Red cell distribution width (≤14.6%) |
| 8. High blood pressure | 33. Lactate dehydrogenase (≤190 U/L) |
| 9. Diabetes mellitus | 34. Alkaline phosphatase (≤115 U/L) |
| 10. Weak/failing kidneys | 35. Uric acid (M: 240-510, F: 160-430mmol/L) |
| 11.Confusion or inability to remember things | 36. Total calcium (2.0-2.5 mmol/L) |
| 12. Difficulty managing money |  |
| 13. Difficulty stooping, crouching, kneeling |  |
| 14. Difficulty lifting or carrying |  |
| 15. Difficulty walking between rooms on same floor |  |
| 16. Difficulty standing up from an armless chair |  |
| 17. Difficulty getting in and out of bed |  |
| 18. Difficulty dressing yourself |  |
| 19. Difficulty grasping/holding small objects |  |
| 20. Difficulty attending social events |  |
| 21. Self-reported health |  |
| 22. Frequency of healthcare use |  |
| 23. Health compared to 1 year ago |  |
| 24. Overnight hospital stays |  |
| 25. Medications |  |

F = female; M = male

**Supplementary Table S2.** Baseline characteristics of participants

| Characteristic | Level | Olfactory function | | P-value |
| --- | --- | --- | --- | --- |
|  |  | Altered | Normal |  |
| Weighted N (%) |  | 23696333.00 (21.7) | 85450820.00 (78.3) |  |
| Unweighted n (%) |  | 1084 (20.9) | 4108 (79.1) |  |
| Sex (%) | Male | 11539856.0 (48.7) | 38108717.0 (44.6) | 0.069 |
|  | Female | 12156477.0 (51.3) | 47342103.0 (55.4) |  |
| Age (years) |  | 56 (49, 64) | 55 (47, 64) | 0.053 |
| Age groups (y) (%) | <65 | 6353565.0 (26.8) | 26857871.0 (31.4) | 0.034 |
|  | 65-80 | 16011757.0 (67.6) | 54523928.0 (63.8) |  |
|  | ≥80 | 1331011.0 ( 5.6) | 4069021.0 ( 4.8) |  |
| BMI (kg/m²) |  | 30.03 (7.18) | 29.10 (6.43) | 0.010 |
| BMI groups (%) | Severely obese | 4838010.0 (20.4) | 12282361.0 (14.4) | 0.030 |
|  | Obese | 5673592.0 (23.9) | 19697029.0 (23.1) |  |
|  | Overweight | 7574874.0 (32.0) | 31176012.0 (36.5) |  |
|  | Underweight/healthy weight | 5609857.0 (23.7) | 22283357.0 (26.1) |  |
| Race and ethnicity (%) | Non-Hispanic White | 18046825.0 (76.2) | 61760112.0 (72.3) | 0.020 |
|  | Other Race | 5649508.0 (23.8) | 23690708.0 (27.7) |  |
| Drinking status (%) | Non-drinkers | 4537211.0 (19.1) | 13113564.0 (15.3) | 0.050 |
|  | Low-frequency drinkers | 8546628.0 (36.1) | 28219182.0 (33.0) |  |
|  | Moderate-frequency drinkers | 3168722.0 (13.4) | 13421332.0 (15.7) |  |
|  | High-frequency drinkers | 7443772.0 (31.4) | 30696742.0 (35.9) |  |
| PA (%) | Inactive | 10379729.0 (43.8) | 35786102.0 (41.9) | 0.466 |
|  | Active | 13316604.0 (56.2) | 49664718.0 (58.1) |  |
| Smoking status (%) | Smoker | 13145429.0 (55.1) | 39388923.0 (46.1) | 0.002 |
|  | Non-smoker | 10650904.0 (44.9) | 46061897.0 (53.9) |  |
| Sleep health (%) | Short sleep | 4166452.0 (17.6) | 11780507.0 (13.8) | 0.137 |
|  | Suboptimal sleep | 6184882.0 (26.1) | 24481761.0 (28.7) |  |
|  | Normal sleep | 13344999.0 (56.3) | 49188552.0 (57.6) |  |

PA, physical activity

**Supplementary Table S3.** Logistic regression analysis of frailty by olfactory function among smokers

|  | Model 1 ^†^ | | Model 2 ^‡^ | | Model 3 ^§^ | |
| --- | --- | --- | --- | --- | --- | --- |
|  | OR(95%CI) | P | OR(95%CI) | P | OR(95%CI) | P |
| Subgroup2 | 1.0 [Reference] |  | 1.0 [Reference] |  | 1.0 [Reference] |  |
| Subgroup4 | 1.48 (1.22–1.79) | **<.001** | 1.39(1.07–1.798) | **<.015** | 1.41(1.09–1.81) | **<.011** |

Subgroup 2 is normal olfactory function & smoker; subgroup 4 is altered olfactory function & smoker. ^†^ Model 1 without adjustments. ^‡^ Model 2 additionally adjusted for sex (male, female), age (year), race (non-Hispanic White, other), body mass index (kg/m²). ^§^ Model 3 additionally adjusted for sleep health (short sleep, suboptimal sleep, normal sleep), drinking status (non-drinker, low-frequency drinker, moderate-frequency drinker, high-frequency drinker) and physical activity (inactive, active).

**Supplementary Table S4.** Logistic regression analysis of frailty by smoking status among participants with altered olfactory function

|  | Model 1 ^†^ | | Model 2 ^‡^ | | Model 3 ^§^ | |
| --- | --- | --- | --- | --- | --- | --- |
|  | OR(95%CI) | P | OR(95%CI) | P | OR(95%CI) | P |
| Subgroup3 | 1.0 [Reference] |  | 1.0 [Reference] |  | 1.0 [Reference] |  |
| Subgroup4 | 1.190 (0.92–1.53) | =.175 | 1.281(0.94–1.75) | =.116 | 1.118(0.80–1.57) | =.503 |

Subgroup 3 is altered olfactory function & non-smoker; subgroup 4 is altered olfactory function & smoker. ^†^ Model 1 without adjustments. ^‡^ Model 2 additionally adjusted for sex (male, female), age (year), race (non-Hispanic White, other), body mass index (kg/m²). ^§^ Model 3 additionally adjusted for sleep health (short sleep, suboptimal sleep, normal sleep), drinking status (non-drinker, low-frequency drinker, moderate-frequency drinker, high-frequency drinker) and physical activity (inactive, active).
